# GPT-4 outperforms junior expert physical therapists in sports medicine rehabilitation: an evaluation of AI response quality and adaptiveness

**DOI:** 10.3389/fresc.2026.1853016

**Published:** 2026-06-09

**Authors:** Eric Hamrin Senorski, Ramana Piussi, Janina Kaarre, Robert Feldt, Kate E. Webster, Martin Hägglund, Rebecca Hamrin Senorski, Johan Högberg, Yinan Yu, Kristian Samuelsson

**Affiliations:** 1 Unit of Physiotherapy, Department of Health and Rehabilitation, Institute of Neuroscience and Physiology, Sahlgrenska Academy, University of Gothenburg, Gothenburg, Sweden; 2 Sahlgrenska Sports Medicine Center, Gothenburg, Sweden; 3 Department of Orthopaedics, Institute of Clinical Sciences, Sahlgrenska Academy, University of Gothenburg, Gothenburg, Sweden; 4 Department of Orthopaedic Surgery, UPMC Freddie Fu Sports Medicine Center, University of Pittsburgh, Pittsburgh, PA, United States; 5 Department of Computer Science and Engineering, Chalmers University of Technology and University of Gothenburg, Gothenburg, Sweden; 6 School of Allied Health, Human Services and Sport, La Trobe University, Melbourne, Australia; 7 Department of Health, Medicine and Caring Sciences, Sport Without Injury ProgrammE (SWIPE), Linköping University, Linköping, Sweden; 8 Department of Health, Medicine and Caring Sciences, Linköping University, Linköping, Sweden; 9 Department of Orthopaedics, Sahlgrenska University Hospital, Mölndal, Sweden

**Keywords:** artificial intelligence, clinical competence, physical therapy, physical therapy modalities, physiotherapy

## Abstract

**Background:**

Artificial intelligence (AI), particularly large language models (LLMs) like ChatGPT, has demonstrated potential in healthcare applications, but its effectiveness in clinical rehabilitation contexts remains underexplored. This study investigated whether GPT-4 (accessed through its online interface ChatGPT) can deliver high-quality, adaptive responses in sports physical therapy comparable to or surpassing those of human professionals.

**Methods:**

Fifty-three sports physical therapy questions were developed by senior experts and answered by GPT-4 and three junior expert physical therapists (JEPs). Responses were tailored for different target audiences: patients, physical therapists, and expert physical therapists. GPT-4 was prompted using structured engineering techniques. A blinded panel of three senior physical therapists/researchers assessed responses for quality and adaptiveness, and identified which responses were superior.

**Results:**

Across all target audiences, GPT-4 outperformed JEPs in both quality and adaptiveness of responses (*p* < 0.001). For responses aimed at patients, GPT-4 was rated best in 26 (55%) questions. For responses aimed at physical therapists, GPT-4 was rated best in 34 (64%) questions. Performance varied by topic, but GPT-4 consistently provided more expert-adapted and contextually appropriate information. GPT-4's responses were especially superior in areas like pain, osteoarthritis, and anterior cruciate ligament rehabilitation.

**Conclusion:**

This study demonstrated that GPT-4 is capable to generate high-quality, adaptive responses in the field of orthopedic sports physical therapy, which can surpass the performance of JEPs in a controlled setting.

## 1 Introduction

The advent of artificial intelligence (AI) has significantly transformed medical and health sciences. Artificial intelligence technologies, particularly large language models (LLMs) such as GPT (through its online interface ChatGPT), have shown immense potential to mimic human-like conversation and provide expert-level information. These models are designed to understand and generate human language with high accuracy and coherence through sophisticated neural network architectures (1). Recent advancements in LLMs have demonstrated their capacity to not only process vast amounts of information but also to provide insights that are comparable to those of human experts (2). The application of AI can potentially revolutionize patient care by offering timely and precise medical advice, support clinical decision-making, and improve the efficiency of healthcare delivery (1–3). However, the reliability and adaptability of AI-generated responses, especially in specialized fields like sports physical therapy, require thorough investigation to ensure their effectiveness and safety. In the area of physical therapy and rehabilitation, AI applications are emerging but are still relatively underexplored. A study by Sumner et al. (4), systematically reviewed the use of AI to create personalized exercise programs for patients undergoing rehabilitation and reported improvements in patient adherence and outcomes. Further, Jang et al. (5), explored the use of machine learning algorithms to predict patient outcomes in orthopedic research and highlighted the potential for AI to enhance personalized care in orthopedics/sports physical therapy. Despite these advancements, there remains a paucity of research that has compared AI-generated advice with human expert recommendations in sports medicine physical therapy.

This study aimed to determine whether GPT-4 (accessed through its online interface ChatGPT) can deliver high-quality, adaptive responses in sports physical therapy comparable to or surpassing those of human professionals.

## 2 Methods

### 2.1 Data source

Three sports medicine rehabilitation experts, all qualified through more than 10 years' experience of clinical and/or research in orthopedic sports medicine physical therapy, were asked to create approximately 53 questions relevant for the field of sports medicine physical therapy. The questions were created and grouped according to different subjects important in sports medicine physical therapy: warm-up, stretching, strength training, core muscle training, asymmetries, technique, body weight, training load, previous injury, pain, osteoarthritis (OA), anterior cruciate ligament (ACL), medial collateral ligament (MCL), and meniscus. For the complete list of these questions, please see Table 1.

**Table 1:** Complete list of 53 questions, divided by subject.

**Warm-up**
1. What is the role of warm up for preventing injuries?
2. What is the optimal time and type of warm up?
3. Why is warm up important?
**Stretching**
4. What is the role of range of motion/stretching on injury prevention?
5. What are the positive effects of stretching?
6. What is the optimal type, dose and time of stretching?
7. When should stretching be performed?
8. What is the role of stretching for sports performance?
**Strength training**
9. What is the role of strength training for injury prevention?
10. What is the optimal frequency and intensity of strength training for injury prevention?
11. What is the role of strength training in rehabilitation?
12. What is the role of strength training for economy in running/cycling?
13. What is the role of strength training for sports performance?
**Core muscle training**
14. What is the role of core muscle training for injury prevention?
15. What is the role of core muscle training for low back pain?
16. What is the optimal type, dosage and intensity of core muscle training?
17. What is the role of core muscle training/strength for sports performance?
18. When is core muscle training recommended in rehabilitation and what is the evidence of its efficacy?
**Asymmetries**
19. How do anatomical asymmetries (bone length, muscle cross section and dimensions etc.) affect injuries? And how can the risks be minimized?
20. How do biomechanical asymmetries (stride length, muscle length imbalances, muscle timing, foot mechanics etc.) affect injuries? And how can the risks be minimized?
21. How do muscle strength asymmetries affect the risk of injury?
22. How do functional asymmetries (e.g., hop tests) affect the risk of injury?
**Technique**
23. How does task performance patterns/technique during exercises/rehabilitation affect the risk of injury?
24. How are improper running biomechanics (e.g., excessive foot drop, step asymmetry and excessive knee valgus) associated with injury?
25. What are the most effective running re-training interventions to reduce the risk of injury?
26. What technique modifications (e.g., landing from jumping without valgus, eliminating excessive hip internal rotations) are effective to reduce injury? How well do the modifications work?
27. Can a biomechanical assessment of an athlete's technique help identify patterns that increase the risk of injury?
**Body weight**
28. How is body weight and BMI associated with the risk of injury?
**Training load**
29. How are sudden increases in training loads associated with the risk of injury?
30. What is the optimal increase in training load without increasing the risk of injury?
31. How is training load associated with injury risk?
32. What is the optimal method to quantify training load?
**Previous injury**
33. How is the history of injury associated with future injury risk?
**Pain**
34. How should pain during exercise/rehabilitation be interpreted?
35. Is pain during rehabilitation acceptable? If so, how much pain can a patient accept without an increased risk of additional damage/injury?
36. How should increase joint swelling during exercise/rehabilitation be interpreted?
37. What is the optimal method to quantify pain during rehabilitation?
38. What is the relationship between pain and tissue damage?
**Osteoarthritis**
39. What is the optimal treatment for osteoarthritis?
40. How effective is exercise therapy for the treatment of osteoarthritis?
41. What type of exercise is recommended for the treatment of osteoarthritis? How well does it work?
42. How effective is weight loss for the treatment of osteoarthritis?
**Anterior cruciate ligament injury**
43. What are the optimal criteria for return to sport after ACL reconstruction?
44. What tests should be used to determine return to sport after ACL reconstruction?
45. What cut-offs for return to sport tests after ACL reconstruction should be used to ensure a safe return to sport, i.e., with minimal risk of second knee injury?
46. When can a patient start running after ACL reconstruction? What criteria should be used?
**Medial collateral ligament injury**
47. What are the indications for using a brace as part of treatment after medial collateral ligament injury? When and how should bracing be used?
48. What is the optimal treatment for medial collateral ligament injuries?
49. What type of exercise therapy is advised after medial collateral ligament injury?
50. When can an athlete return to sport after medial collateral ligament injury?
**Meniscus rupture**
51. What is optimal non-surgical treatment for meniscus rupture? Are there any differences in the recommendation based on the type of rupture, location and size of the meniscus rupture?
52. When is bracing advised as part of treatment after meniscus rupture? How shall bracing be used?
53. What is the optimal exercise therapy for the treatment of meniscus rupture?

**Figure 1:**
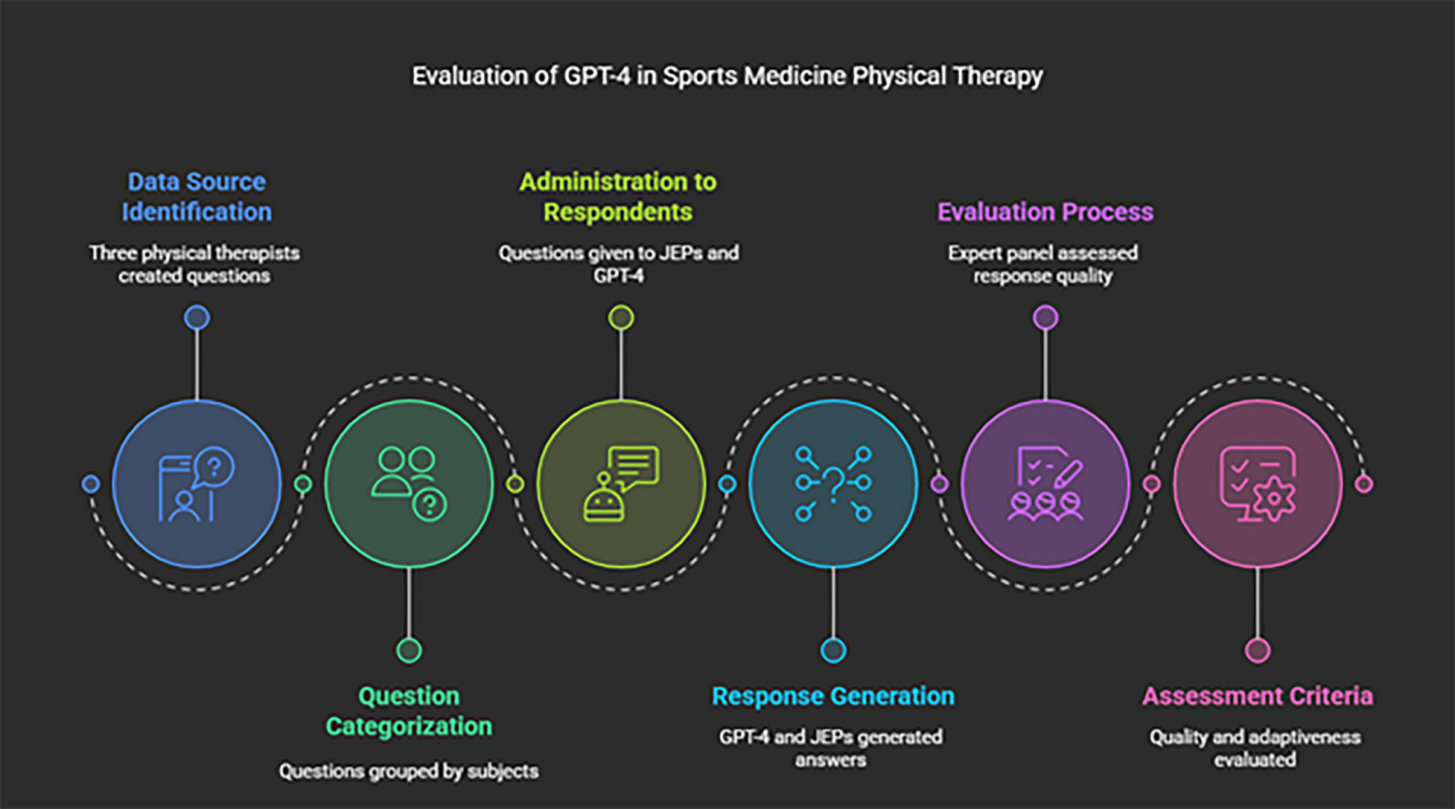
Overview of study execution. JEP, junior expert physical therapists.

The questions were divided and administered to three junior expert physical therapists (JEP). The JEP were two males and one female physical therapists, with degrees from three different Universities, aged 29–35 years at time of questions completion. All JEP were PhD students at the time of questions completion, and worked in the same orthopedic sports rehabilitation clinic. The JEPs had approximately 6–8 years of clinical experience at the time of administration. All questions were also administered to GPT-4. Both JEPs and GPT-4 were asked to respond to the questions twice: first with response adapted to patient, and second with the response adapted to a physiotherapist. In addition, GPT-4 was asked to respond to the questions adapted to an expert physical therapist. A panel of three rehabilitation experts (EHS, KW, and MH) judged responses to all the questions for quality and adaptiveness, see Figure 1 for overview of the study execution.

### 2.2 GPT-4

In this study, we utilized GPT-4 (accessed through its online interface ChatGPT), a conversational AI system based on LLMs that employs a Transformer-style neural network architecture. GPT was initially introduced as a research preview in November 2022 (6). The version used in this study is based on the GPT-4 model, introduced in March 2023 (7), which has been reported to be capable of generating responses with human-like qualities, displaying early signs of general intelligence (8).

### 2.3 Prompting and data collection

The effectiveness of GPT-4 is significantly influenced by the way prompts are designed. In this current study, the principles of “prompt engineering”, which is a specialized field that provides valuable guidance for creating effective prompts, were followed (9, 10). Created prompts were used to allow optimal responses from GPT-4, instructing it to assume the role of an expert physical therapist and provide responses based on the most recent research findings and best clinical practices in the field. Detailed instructions were given to define the target groups (patient, physical therapist, and expert physical therapist) and specify their expected knowledge level. Furthermore, the length of the responses was restricted to ensure a feasible assessment process. Considering that the target group of physical therapists and expert physical therapists frequently use more precise terminology and concepts, slightly longer responses (up to seven sentences instead of five sentences) were permitted to these target groups compared to the patient target group. Table 2 presents the specific prompts.

**Table 2:** Specific prompts.

Specifics	Target group		
	Patient	Physiotherapist	Expert physiotherapist
General	Your task is to answer questions about rehabilitation and injury prevention. I will write questions to you, and you will answer based on the latest, state-of-the-art knowledge and on current established standards for treatment. I want you to only reply with your brief answer, nothing else. Your main goal is that your answers are correct (in line with the latest knowledge), as complete as possible (cover the key information), and adapted to the target group.	Your task is to answer questions about rehabilitation and injury prevention. I will write questions to you, and you will answer based on the latest, state-of-the-art knowledge and on current established standards for treatment. I want you to only reply with your brief answer, nothing else. Your main goal is that your answers are correct (in line with the latest knowledge), as complete as possible (cover the key information), and adapted to the target group.	Your task is to answer questions about rehabilitation and injury prevention. I will write questions to you, and you will answer based on the latest, state-of-the-art knowledge and on current established standards for treatment. I want you to only reply with your brief answer, nothing else. Your main goal is that your answers are correct (in line with the latest knowledge), as complete as possible (cover the key information), and adapted to the target group.
Target	The target group is a patient that is an adult that has no specific medical education, training, or experience.	The target group is a physiotherapist that has 3–4 years of education in physical therapy and has a maximum of 2 years of clinical experience in rehabilitation.	The target group is an expert physiotherapist that has 3–4 years of education in physical therapy as well as long clinical and research experience in rehabilitation as well as a deep understanding of the specific treatment options and their relative merits.
Contexts	Your answers need to be understandable and rather brief, preferably 2–3 sentences and not longer than 5 sentences. Please do not use overly complex language or wording: the goal is to be clear, direct, and understandable. You cannot assume the patient has deep knowledge of anatomy, rehabilitation or exercise physiology, nor about the jargon or specific terms of the field, but you can assume that the patient has a basic understanding of the human body and its functions.	You can assume the physiotherapist has basic knowledge of anatomy, rehabilitation, and exercise physiology but has no deeper knowledge about the specific treatment options and their relative merits to rehabilitation and injury prevention.	Your answers need to be precise but rather brief, preferably 2–3 sentences and not longer than 7 sentences. You can use complex language and wording: the goal is to be precise, give expert advice, and provide a broad sense of multiple treatment options. Your answers should be as complete as possible and not leave out any of the important factors. You can assume the expert physiotherapist has deep knowledge of anatomy, rehabilitation, and exercise physiology as well as a deep understanding of evidence-based medicine, specific treatment options as well as their relative merits to rehabilitation and injury prevention.

### 2.4 Turing test and assessment

The sequence of questions was randomized for all three target groups to minimize the influence of context and order. The three JEPs, with at least five years of clinical experience, were instructed to respond to the same set of questions as GPT-4, following the same randomized order. Each JEP was asked to respond to 17 or 18 questions and provide responses separately for both patients and physical therapists, with the same specifications as GPT-4 regarding target population and answers length. The purpose of this approach was to ensure that the panel of three experts remained unaware of the identity of the responders and to facilitate a comparison of response quality between GPT-4 and the JEPs (11).

After response collection, a list of responses was created, including the original questions, GPT-4's response, and the JEPs' responses. This list of responses was subsequently reviewed by the panel of three experts. Each expert physical therapist/researcher was presented with the complete list of responses, the JEP's response, and GPT-4's response. The responses were labeled as “1” and “2”, to maintain unawareness to the identity of the responders, i.e., JEP or GPT-4. The list of responses was designed to assess the quality and adaptiveness of the responses. The quality rating was intended to reflect not only correctness and completeness of the content, but also clinical relevance and safety. Expert assessors were instructed to consider whether the response was appropriate and safe for the intended audience, particularly in the context of rehabilitation advice. Quality was rated as follows: 0 = very poor, 1 = poor, 2 = acceptable, 3 = good, or 4 = very good (12). With adaptiveness of the responses is meant how well-adapted the response was to the target population (0 = not adapted, 1 = slightly adapted, 2 = moderately adapted, 3 = adapted, and 4 = very well adapted). This quality rating has been used in previous publications (13, 14). Furthermore, the assessors were asked to determine which of the responses provided was better, without them knowing whether the response was provided by GPT-4 or the JEPs. This was done for both the “patient” and “physical therapist” target groups. Before assessing the responses, the assessors participated in a 1 h calibration meeting to discuss the rating criteria in detail and pilot-test the assessment procedure using examples.

### 2.5 Follow-up comparison with GPT-4o

Following the conclusion of the primary assessment, GPT-4 was no longer accessible via the ChatGPT interface, as of 2025-04-30, and had been replaced by GPT-4o. To explore potential performance improvements, we identified the five questions with the lowest mean quality ratings from the original GPT-4 responses. These questions were re-entered into ChatGPT using GPT-4o, prompted using the same structured approach and response instructions as in the original study. The resulting GPT-4o responses were then evaluated in a blinded format by the same expert panel, using identical rating procedures for information quality. Experts also indicated which of the two responses—GPT-4 or GPT-4o—they judged to be superior for each question. Mean and median quality scores were calculated, and comparative judgments were summarized.

### 2.6 Statistical analysis

For each question, all three expert assessors independently judged which of the two responses (GPT-4 vs. JEP) was better. These individual judgments were recorded separately. Table 3 presents a summary of how many experts (0 to 3) rated GPT-4 or JEP as providing the better response for each question. Thus, the table reflects agreement patterns among the three experts for each question. Response ratings, that is, information quality and adaptiveness were analyzed using paired Wilcoxon signed-rank tests to compare scores between GPT-4 and JEPs. The tests were performed separately for each target group: patients and physical therapists. For each criterion, the null hypothesis was that there was no difference in median scores between the two response sources. Response ratings were analyzed using the paired Wilcoxon signed-rank test, which evaluates differences in medians, in addition, mean values are reported in the results to facilitate interpretability and comparison across groups. The Wilcoxon test was chosen due to the ordinal nature of the Likert-scale data and the non-normal distribution of scores. Inferential comparisons between GPT-4 and JEPs were performed only for patient-targeted and physical therapist-targeted responses, where matched GPT-4 and JEP responses were available. Responses tailored to expert physical therapists were generated by GPT-4 only and were therefore summarized descriptively, without comparative inferential testing. Inter-rater agreement for the forced-choice judgment of which response was superior was assessed using Fleiss’ kappa, because three independent expert assessors evaluated each response pair. Agreement was calculated separately for patient-targeted and physical therapist-targeted responses. The significance level was set at 95%.

**Table 3:** Number of questions in which ChatGPT or JEPs provided the best answers.

Number of experts rating the response was best ChatGPT vs. JEP	ChatGPT	JEP
Response to patients		
3–0	26	
2–1	17	
1–2		7
0–3		3
Response to physiotherapists		
3–0	34	
2–1	14	
1–2		5
0–3		0

JEP, junior expert physiotherapist.

## 3 Results

**Figure 2:**
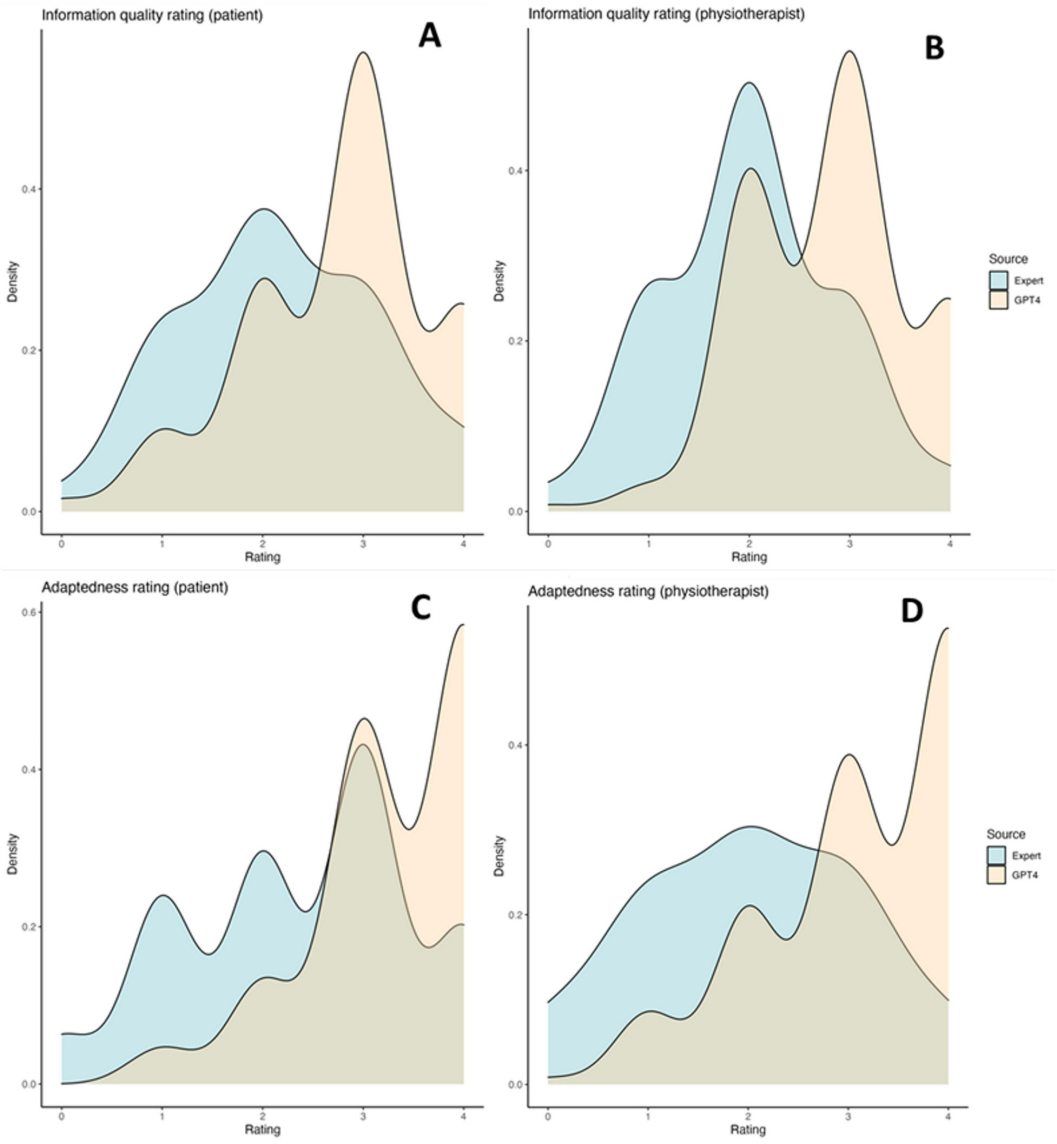
Summary of response ratings for information quality and adaptedness. Blue area: junior expert physiotherapist responses; orange area: GPT-4 responses. **(A)** quality in answers to patients; **(B)** quality in answers to physical therapists; **(C)** adaptedness in answers to patients; **(D)** adaptedness in answers to physical therapists.

In the responses adapted to patients, GPT-4 scored significantly higher than JEPs for information quality (median 3, mean: 2.774 vs. median 2, mean 2.182, *p* ≤ 0.001), and adaptiveness (median 4, mean: 3.296 vs. median 3, mean 2.384, *p* ≤ 0.001) (Table 4 and Figure 2). In the responses adapted to physical therapists, GPT-4 outperformed JEPs in both information quality (median 3, mean: 2.805 vs. median 2, mean 2.025, *p* ≤ 0.001), and adaptiveness (median 3.25, mean: 3.113 vs. median 2.50, mean 2.025, *p* ≤ 0.001) (Table 4 and Figure 2).

**Table 4:** Median and mean response ratings for quality and adaptiveness between JEPs and GPT-4.

Responses	Groups	Quality Median (mean)	*p*-value	Adaptiveness Median (mean)	*p*-value
Response to patients	GPT-4	3 (2.774)	≤0.001	4 (3.296)	≤0.001
	JEP	2 (2.182)		3 (2.384)	
Response to physical therapists	GPT-4	3 (2.805)	≤0.001	3.25 (3.113)	≤0.001
	JEP	2 (2.025)		2.50 (2.025)	
Response to expert physical therapists	GPT-4	3 (3.097)		3 (3.221)	

JEP, junior expert physiotherapist.

The three experts agreed that GPT-4 provided the best responses to patients in 26 questions, while JEPs provided the better responses in 3 questions: questions number 23 (How does task performance patterns/technique during exercises/rehabilitation affect the risk of injury?), number 27 (Can a biomechanical assessment of an athlete's technique help identify patterns that increase the risk of injury?) and number 50 (When can an athlete return to sport after MCL injury?). The experts agreed that GPT-4 provided the best responses to physiotherapists in 34 questions, while JEPs never provided the best answers (Table 3). Inter-rater agreement for the forced-choice judgments showed observed agreement of 69.8% for patient-targeted responses and 76.1% for physical therapist-targeted responses. The corresponding Fleiss’ kappa values were 0.20 and 0.07, respectively. The lower kappa values relative to observed agreement should be interpreted in light of the skewed distribution of judgments, where GPT-4 was preferred in most comparisons.

When the responses to questions were stratified according to the 14 sports medicine subjects, GPT-4's responses had higher ratings for quality or adaptiveness, compared to responses from the JEPs (Tables 5, 6).

**Table 5:** Median ratings for response to patients for quality and adaptiveness for GPT-4 and JEPs.

Subject	Quality; median (mean)		Adaptiveness; median (mean)	
	GPT-4	JEPs	GPT-4	JEPs
Warm-up	3 (2.67)	2 (2.11)	3 (3.22)	2 (2.22)
Stretching	2 (1.93)	2 (1.87)	3 (3.13)	2 (2.20)
Strength training	3 (3.13)	2 (2.47)	3 (3.47)	2 (2.60)
Core strength training	3 (2.47)	2 (1.87)	3 (3.07)	2 (2.07)
Asymmetries	2.5 (2.33)	2.5 (2.50)	3 (3.00)	1.5 (1.83)
Technique	2 (2.27)	2 (2.13)	3 (2.73)	3 (2.60)
Body weight	3 (3.00)	2 (2.00)	3 (3.33)	3 (2.67)
Training load	3 (2.92)	2.5 (2.25)	4 (3.42)	3 (2.50)
Previous injury	3 (3.00)	2 (1.67)	4 (3.67)	2 (1.67)
Pain	3 (3.33)	2 (2.40)	3 (3.80)	2 (3.13)
OA	3 (3.33)	2 (2.00)	3 (3.67)	1 (2.08)
ACL	3 (3.08)	3 (2.75)	3.5 (3.33)	3 (2.67)
MCL	3 (3.00)	2 (1.92)	4 (3.67)	2 (2.33)
Meniscus	3 (2.89)	2 (2.11)	3 (2.89)	3 (2.22)

ACL, anterior cruciate ligament; MCL, medial collateral ligament; JEPs, junior expert physical therapists; OA, osteoarthritis.

**Table 6:** Median ratings for response to physical therapists for quality and adaptiveness for GPT-4 and JEPs.

Subject	Quality; median (mean)		Adaptiveness; median (mean)	
	GPT-4	JEPs	GPT-4	JEPs
Warm-up	3 (2.78)	2 (1.67)	3 (3.00)	2 (1.89)
Stretching	2 (2.13)	2 (2.13)	3 (2.93)	2 (2.07)
Strength training	3 (3.13)	3 (2.47)	3 (3.07)	3 (2.40)
Core strength training	3 (2.47)	2 (1.73)	3 (2.93)	2 (1.93)
Asymmetries	2 (2.33)	1 (1.58)	3 (2.92)	1.5 (1.50)
Technique	3 (2.73)	1 (1.53)	3 (3.20)	2 (1.67)
Body weight	2 (2.33)	2 (2.67)	3 (2.33)	2 (2.33)
Training load	3 (3.00)	2 (2.00)	4 (3.42)	2 (2.08)
Previous injury	3 (2.67)	2 (2.00)	4 (3.33)	1 (1.33)
Pain	3 (3.47)	3 (2.53)	4 (3.40)	3 (2.53)
OA	3 (3.17)	2 (2.08)	3.5 (3.17)	1 (1.75)
ACL	3 (3.08)	2 (2.25)	4 (3.42)	2 (2.17)
MCL	3 (2.67)	2 (1.75)	3.5 (3.17)	2 (1.92)
Meniscus	3 (2.89)	2 (2.33)	3 (2.78)	3 (2.44)

ACL, anterior cruciate ligament; MCL, medial collateral ligament; JEPs, junior expert physical therapists; OA, osteoarthritis.

In response to patients, the JEP's highest quality response was given in question number 20 (How do biomechanical asymmetries (stride length, muscle length imbalances, muscle timing, foot mechanics etc.) affect injuries? And how can the risks be minimized?) and number 23 (How does task performance patterns/technique during exercises/rehabilitation affect the risk of injury?), where JEPs received a quality rating of 3.33 (mean) and 3 (median). In comparison, GPT-4 received scores of 3.67 and 2.33 (mean) 4 and 2 (median) for responses to questions number 20 and 23, respectively (Supplementary File S1).

In the responses adapted to expert physical therapists, GPT-4 had consistently higher ratings across most categories, with median quality scores of 3 or above in 12 of the 14 subject areas (Table 7). The highest quality score was observed in the body weight category (mean 3.67, median 4), while the lowest was seen in the meniscus category (mean 2.44, median 2) (Supplementary File S2).

**Table 7:** Median ratings for response to expert physical therapists for quality and adaptiveness for GPT-4 only.

Subject	Quality; median (mean)	Adaptiveness; median (mean)
Warm-up	3 (2.89)	3 (3.11)
Stretching	3 (2.47)	3 (3.07)
Strength training	4 (3.60)	3 (3.40)
Core strength training	3 (2.87)	3 (3.07)
Asymmetries	3 (3.08)	3 (3.08)
Technique	3 (2.73)	3 (3.07)
Body weight	4 (3.67)	3 (3.00)
Training load	3 (3.08)	3 (3.25)
Previous injury	4 (3.33)	4 (3.33)
Pain	4 (3.20)	4 (3.47)
OA	3.5 (3.33)	4 (3.50)
ACL	4 (3.42)	4 (3.58)
MCL	3 (3.25)	3 (3.17)
Meniscus	2 (2.44)	3 (3.00)

ACL, anterior cruciate ligament; MCL, medial collateral ligament; JEPs, junior expert physical therapists; OA, osteoarthritis.

### 3.1 Follow-up comparison with GPT-4o

The three expert raters consistently judged the new responses generated by GPT-4o to be of higher quality than the original GPT-4 responses. Mean and median quality scores improved for all five questions, except for question 7, response to physiotherapists: “How are improper running biomechanics (e.g., excessive foot drop, step asymmetry, and excessive knee valgus) associated with injury?”, where the mean and median scores were unchanged, but all three experts still rated the GPT-4o response as superior. Detailed results are presented in Table 8.

**Table 8:** Ratings for responses to questions with the overall lowest quality in the response from GPT-4.

Question number (target group)	Median (mean) response GPT-4	Median (mean) response GPT-4o	Preferred response GPT-4o vs. GPT-4
30 (patient)	1 (1.33)	2.7 (3)	3–0
33 (patient)	1 (1.33)	3 (3)	3–0
52 (patient)	2 (1.67)	3 (3)	3–0
7 (physiotherapist)	2 (2)	2 (2)	3–0
28 (physiotherapist)	2 (1.67)	3 (3)	3–0

## 4 Discussion

The main finding of this study is that GPT-4 outperformed JEPs in both the quality and adaptiveness of responses to sports physical therapy-related questions. This suggests that LLMs, such as GPT-4, hold the potential to provide qualitative and adaptive clinical advice in rehabilitation settings, that could meet or exceed the quality and adaptiveness of advice provided by trained junior professionals rehabilitation experts, and, thereby, carries the opportunity to a more accessible healthcare.

Our findings align with previous studies investigating the use of AI in rehabilitation and patient care (15–17). The use of machine learning algorithms to predict clinically meaningful outcomes in orthopedic care has shown promising potential to support more tailored treatments in physical therapy (5). In addition, a systematic review of AI in physical rehabilitation suggested that personalized exercise programs generated by AI may improve both patient adherence and outcomes (4).

The performance of GPT-4 in this study underline the broader applicability of AI in personalized medical advice, specifically in areas such as sports medicine physical therapy.

GPT-4's ability to synthesize vast amounts of clinical and scientific knowledge likely contributed to its superior performance in this study. GPT-4 is likely capable to assess and process the latest peer-reviewed literature, which allows it to provide accurate, evidence-based responses (18, 19). On the other hand, we do not know whether GPT can accurately weight the evidence it bases responses upon. A case study and a systematic review have different potential to impact decisions, given the risk of bias, on a clinician's choice of information to provide. Future studies need to specifically analyze how well GPT can understand and value the concept of level of evidence.

The adaptability of GPT-4's responses to different target audiences—patients, junior physical therapists, and expert physical therapists—reflects the model's strength in tailoring information according to the user's knowledge level. The adaptability of LLMs may be especially relevant for patient education, rehabilitation adherence, triage, exercise instruction, and tele-rehabilitation, where information often needs to be individualized and communicated remotely. In these contexts, AI could support clinicians by translating rehabilitation concepts into patient-friendly language, reinforcing adherence strategies, providing standardized exercise explanations, and helping identify situations that require further clinical assessment (20, 21). However, these applications require clinical oversight, safety validation, and clear escalation pathways to qualified healthcare professionals. This potential role is supported by work in low back pain education (22), where ChatGPT-4.0 outperformed ChatGPT-3.5 in response quality and reliability, although both models showed limitations when addressing psychosocial aspects of care. The implementation of AI-generated rehabilitation recommendations also raises important ethical and medicolegal considerations. In patient-facing or clinician-support contexts, responsibility for clinical decisions must remain clearly defined, particularly when AI-generated advice is used to inform triage, exercise progression, or recommendations to seek care (23). Issues related to informed consent, data privacy, algorithmic bias, transparency, and accountability should therefore be addressed before LLMs are integrated into routine rehabilitation practice. In this context, AI should be regarded as a supportive tool rather than an autonomous clinical decision-maker. While the quality ratings included an implicit assessment of clinical safety, it is important to note that our evaluation was limited to written content and did not include systematic safety validation. Future studies should explicitly examine the safety of AI-generated recommendations in dynamic, real-world rehabilitation contexts. GPT´s superiority over JEPs may partly be explained by the fact physical therapists seldom provide advice in written form. Additionally, caution is warranted when generalizing our findings to physiotherapists at large. The responses were provided by three JEP with specific training and clinical experience in sports medicine rehabilitation. While their backgrounds make them appropriate comparators for GPT-4 in this context, the results may not fully represent the broader physiotherapy population, particularly across different educational systems, clinical roles, or levels of experience. Nevertheless, GPT-4's highly rated adaptability is consistent with studies on prompt engineering that suggest LLMs can be fine-tuned to respond appropriately to varied prompts, to improve both clarity and accuracy (24). It should not be understated that the high adaptability does require knowledge about prompt engineering from the user. A patient with no specific knowledge about prompt engineering might struggle to receive responses adapted to him/her (25, 26).

## 5 Limitations

Despite promising results from this present study, several limitations must be acknowledged. First, while GPT-4 outperformed JEPs in terms of quality and adaptiveness, this study was conducted in a controlled environment where the responses were evaluated based solely on written communication. In clinical practice, physical therapists rely on real-time interactions, including non-verbal cues, patient feedback, and physical assessments, which AI currently cannot replicate (12). Therefore, while GPT-4 demonstrated strong performance in text-based assessments, its utility in live, dynamic clinical environments remains untested. Another limitation is the AI model's knowledge cutoff.

A further limitation is the potential for LLMs to generate recommendations that are plausible and confidently phrased but inaccurate, unsupported, or outdated. Such hallucinated or outdated recommendations may be particularly problematic in rehabilitation contexts where advice regarding loading, progression, return to sport, or referral may have direct consequences for patient safety (27). Therefore, AI-generated rehabilitation recommendations should be interpreted with caution and verified against current evidence, clinical guidelines, and individual patient presentation before being applied in practice.

While GPT-4 can generate responses based on extensive training data, its knowledge is not continuously updated in real time. This means that responses, although always uniquely presented, that is, never the exact same response, may not reflect the most current research or clinical guidelines unless the model is regularly retrained. One limitation of GPT is its black-box architecture, which makes it difficult to trace the specific sources or reasoning behind its responses, potentially limiting transparency and accountability in clinical decision-making. However, continuous education cannot be guaranteed by a physiotherapist. This issue, regardless of whether seeing a physiotherapist using or not using LLMs, is critical in fields such as sports physical therapy, where evidence-based practices are continually evolving. The integration of AI into medical practice necessitates regular updates to ensure the reliability and accuracy of the information provided (2).

Questions were grouped by predefined subjects relevant to orthopedic sports physical therapy, such as “pain” or “anterior cruciate ligament”. However, some questions could arguably align more closely with overarching themes such as injury prevention, risk factors, rehabilitation, or assessment. Future studies may consider alternative thematic frameworks to better reflect clinical reasoning and practice patterns.

The study design inherently favored AI in terms of standardization. Human physical therapists bring a wide range of individual experiences, insights, and subjective decision-making to clinical care. The evaluation process, which compared responses based solely on text, did not account for this variability. Future studies could involve more dynamic assessments, such as real-time consultations or case simulations, to better understand how AI and human physical therapists might work together to optimize patient outcomes. Lastly, the ad-hoc analysis of the five poorest ratings from GPT-4 were re-analysed using GPT-4o and resulted in improved quality ratings and preferred responses, compared to previous responses from GPT-4. This finding suggests that future LLMs may have the capacity to more closely align with current best practice, which could, if appropriately validated and implemented, contribute to democratizing access to evidence-informed physical therapy recommendations.

## 6 Conclusion

This study demonstrated that GPT-4 is capable to generate high-quality, adaptive responses in the field of sports physical therapy, which can surpass the performance of junior expert physical therapists in a controlled setting. Large Language Models like GPT have potential to complement, human sports physical therapy expertise. Further research is needed to assess how AI can be integrated into real-world clinical practice and to explore its role in augmenting sports physical therapy decision making.

## Data Availability

The raw data supporting the conclusions of this article will be made available by the authors, without undue reservation.
