# Supplementary material for: GPT-4 outperforms junior expert physical therapists in sports medicine rehabilitation: an evaluation of AI response quality and adaptiveness
Source: Front Rehabil Sci. 2026 Jun 9;7:1853016. doi: 10.3389/fresc.2026.1853016 (PMC13288665; doi:10.3389/fresc.2026.1853016)
Supplement: Supplementary file 2 [file Supplementaryfile2.docx]

| Supplementary file 2: Responses to questions tailored for physical therapists. | | | | | | | | | | |
| --- | --- | --- | --- | --- | --- | --- | --- | --- | --- | --- |
| Question number | Mean quality GPT | Mean quality JEPs | Mean adaptiveness GPT | Mean adaptiveness JEPs | Median quality GPT | Median quality JEPs | Median adaptiveness GPT | Median adaptiveness JEPs | GPT better | JEPs better |
| 1 | 3.0 | 1.33 | 3.33 | 2.0 | 3.0 | 1.0 | 3.0 | 2.0 | 3 | 0 |
| 2 | 2.33 | 1.33 | 3.0 | 2.0 | 3.0 | 1.0 | 3.0 | 2.0 | 2 | 1 |
| 3 | 3.0 | 2.67 | 2.67 | 3.0 | 3.0 | 3.0 | 3.0 | 3.0 | 1 | 2 |
| 4 | 2.33 | 1.33 | 3.0 | 2.0 | 2.0 | 1.0 | 3.0 | 2.0 | 3 | 0 |
| 5 | 3.67 | 2.0 | 3.0 | 2.0 | 4.0 | 2.0 | 4.0 | 2.0 | 3 | 0 |
| 6 | 3.33 | 2.0 | 3.33 | 2.0 | 3.0 | 2.0 | 4.0 | 1.0 | 3 | 0 |
| 7 | 2.0 | 1.33 | 3.0 | 1.33 | 2.0 | 2.0 | 3.0 | 1.0 | 3 | 0 |
| 8 | 3.0 | 3.0 | 2.67 | 3.0 | 3.0 | 3.0 | 3.0 | 3.0 | 1 | 2 |
| 9 | 2.67 | 2.0 | 3.33 | 1.67 | 3.0 | 2.0 | 3.0 | 2.0 | 3 | 0 |
| 10 | 2.67 | 1.67 | 3.33 | 1.33 | 3.0 | 1.0 | 3.0 | 1.0 | 2 | 1 |
| 11 | 2.33 | 2.33 | 3.0 | 3.0 | 2.0 | 2.0 | 3.0 | 3.0 | 2 | 1 |
| 12 | 2.67 | 2.33 | 3.33 | 3.0 | 3.0 | 2.0 | 4.0 | 3.0 | 2 | 1 |
| 13 | 2.67 | 2.33 | 3.0 | 2.67 | 2.0 | 2.0 | 3.0 | 3.0 | 1 | 2 |
| 14 | 3.33 | 2.33 | 4.0 | 2.67 | 4.0 | 2.0 | 4.0 | 3.0 | 3 | 0 |
| 15 | 3.33 | 3.33 | 3.0 | 3.33 | 3.0 | 3.0 | 3.0 | 3.0 | 2 | 1 |
| 16 | 3.67 | 1.0 | 3.33 | 1.0 | 4.0 | 1.0 | 4.0 | 1.0 | 3 | 0 |
| 17 | 2.67 | 2.0 | 3.33 | 1.33 | 3.0 | 2.0 | 4.0 | 1.0 | 3 | 0 |
| 18 | 3.0 | 1.67 | 3.0 | 1.67 | 3.0 | 2.0 | 3.0 | 2.0 | 3 | 0 |
| 19 | 2.67 | 1.67 | 2.67 | 1.33 | 3.0 | 1.0 | 3.0 | 1.0 | 3 | 0 |
| 20 | 3.33 | 1.67 | 3.0 | 1.67 | 3.0 | 2.0 | 4.0 | 1.0 | 3 | 0 |
| 21 | 3.0 | 2.67 | 3.33 | 2.67 | 3.0 | 3.0 | 3.0 | 3.0 | 3 | 0 |
| 22 | 2.33 | 2.0 | 3.0 | 1.67 | 2.0 | 2.0 | 3.0 | 2.0 | 3 | 0 |
| 23 | 2.67 | 2.33 | 2.67 | 2.33 | 3.0 | 2.0 | 3.0 | 2.0 | 3 | 0 |
| 24 | 2.33 | 2.0 | 2.33 | 2.0 | 2.0 | 2.0 | 3.0 | 2.0 | 1 | 2 |
| 25 | 3.33 | 2.33 | 3.67 | 2.67 | 3.0 | 2.0 | 4.0 | 3.0 | 3 | 0 |
| 26 | 2.33 | 2.0 | 2.67 | 2.33 | 2.0 | 2.0 | 3.0 | 2.0 | 2 | 1 |
| 27 | 3.33 | 1.33 | 3.0 | 1.33 | 3.0 | 1.0 | 3.0 | 1.0 | 3 | 0 |
| 28 | 1.67 | 2.33 | 3.0 | 2.0 | 2.0 | 3.0 | 3.0 | 3.0 | 1 | 2 |
| 29 | 2.67 | 2.0 | 3.33 | 2.0 | 3.0 | 2.0 | 4.0 | 2.0 | 2 | 1 |
| 30 | 2.33 | 1.67 | 2.67 | 2.67 | 2.0 | 2.0 | 2.0 | 3.0 | 2 | 1 |
| 31 | 3.33 | 1.33 | 3.33 | 0.33 | 3.0 | 1.0 | 4.0 | 0.0 | 3 | 0 |
| 32 | 3.67 | 2.0 | 3.33 | 0.67 | 4.0 | 2.0 | 4.0 | 1.0 | 3 | 0 |
| 33 | 2.33 | 2.0 | 3.0 | 1.67 | 2.0 | 2.0 | 3.0 | 2.0 | 3 | 0 |
| 34 | 2.33 | 2.67 | 2.33 | 2.33 | 2.0 | 2.0 | 3.0 | 2.0 | 3 | 0 |
| 35 | 2.67 | 2.33 | 3.0 | 2.33 | 3.0 | 2.0 | 4.0 | 2.0 | 2 | 1 |
| 36 | 2.67 | 1.67 | 3.33 | 2.0 | 3.0 | 2.0 | 3.0 | 1.0 | 2 | 1 |
| 37 | 3.67 | 3.0 | 3.67 | 3.0 | 4.0 | 3.0 | 4.0 | 3.0 | 3 | 0 |
| 38 | 3.67 | 1.67 | 3.33 | 1.67 | 4.0 | 1.0 | 4.0 | 1.0 | 3 | 0 |
| 39 | 2.33 | 1.67 | 2.67 | 2.0 | 2.0 | 2.0 | 3.0 | 2.0 | 2 | 1 |
| 40 | 2.33 | 1.0 | 2.67 | 1.0 | 2.0 | 1.0 | 3.0 | 0.0 | 3 | 0 |
| 41 | 2.33 | 1.0 | 3.33 | 1.0 | 3.0 | 1.0 | 3.0 | 1.0 | 3 | 0 |
| 42 | 2.33 | 2.0 | 3.33 | 3.0 | 2.0 | 2.0 | 3.0 | 3.0 | 3 | 0 |
| 43 | 3.33 | 3.0 | 3.33 | 3.0 | 3.0 | 3.0 | 3.0 | 3.0 | 3 | 0 |
| 44 | 2.0 | 1.67 | 2.67 | 2.0 | 2.0 | 2.0 | 3.0 | 2.0 | 3 | 0 |
| 45 | 3.33 | 2.67 | 3.0 | 2.0 | 4.0 | 3.0 | 4.0 | 2.0 | 3 | 0 |
| 46 | 3.0 | 3.0 | 3.33 | 3.0 | 3.0 | 3.0 | 4.0 | 3.0 | 3 | 0 |
| 47 | 2.67 | 2.0 | 3.33 | 2.0 | 3.0 | 2.0 | 3.0 | 2.0 | 3 | 0 |
| 48 | 3.67 | 2.33 | 4.0 | 1.67 | 4.0 | 2.0 | 4.0 | 1.0 | 3 | 0 |
| 49 | 3.33 | 2.0 | 3.67 | 1.67 | 3.0 | 2.0 | 4.0 | 2.0 | 3 | 0 |
| 50 | 2.67 | 1.33 | 3.67 | 1.0 | 3.0 | 1.0 | 4.0 | 1.0 | 3 | 0 |
| 51 | 3.33 | 3.0 | 3.33 | 2.67 | 3.0 | 3.0 | 4.0 | 2.0 | 2 | 1 |
| 52 | 1.33 | 1.67 | 2.67 | 1.0 | 2.0 | 2.0 | 3.0 | 1.0 | 2 | 1 |
| 53 | 2.67 | 2.33 | 2.67 | 2.67 | 3.0 | 2.0 | 3.0 | 3.0 | 2 | 1 |
| GPT = ChatGPT; JEPs = Junior Expert Physical therapists | | | | | | | | | | |
